# Severe inflammatory disease activity 14 months after cessation of Natalizumab in a patient with Leber’s optic neuropathy and multiple sclerosis – a case report

**DOI:** 10.1186/s12883-016-0720-2

**Published:** 2016-10-18

**Authors:** Trygve Holmøy, Antonie G. Beiske, Svetozar Zarnovicky, Aija Zuleron Myro, Egil Røsjø, Emilia Kerty

**Affiliations:** 1 Department of Neurology, Akershus University Hospital, Lørenskog, Norway; 2 Institute of Clinical Medicine, University of Oslo, Oslo, Norway; 3 Multiple Sclerosis Centre Hakadal, Hakadal, Norway; 4 Department of Radiology, Akershus University Hospital, Lørenskog, Norway; 5 Department of Neurology, Oslo University Hospital Rikshospitalet, Oslo, Norway

**Keywords:** Multiple sclerosis, Leber’s optic neuropathy, Natalizumab, Rebound, Immune reconstitution inflammatory syndrome, Case report

## Abstract

**Background:**

Leber’s hereditary optic neuropathy (LHON) co-occuring with multiple sclerosis-like disease (LHON-MS) is suggested to be a separate disease entity denoted Harding’s disease. Little is known about the response to initiation and discontinuation of potent immunomodulatory treatment in LHON-MS.

**Case presentation:**

We describe a LHON-MS patient with 27 years disease duration who developed severe disease activity peaking 14 months after discontinuation of natalizumab, with extensive new inflammatory lesions throughout the brain and in the spinal cord resembling immune inflammatory reconstitution syndrome. She had previously been clinically and radiologically stable on natalizumab treatment for 6 years, and before that only experienced subtle clinical activity during 9 years on interferon beta1a.

**Conclusion:**

This is the first report on severe exacerbation of inflammatory disease activity after discontinuation of natalizumab in LHON-MS, and suggests that late rebound activity can occur in these patients.

## Background

Leber’s hereditary optic neuropathy (LHON) is caused by point mutations in mitochondrial DNA that encodes complex 1 in the respiratory chain, and usually manifests as subacute severe and painless deterioration of central visual acuity, caused by loss retinal ganglion cells in the papillomacular bundle [1]. The co-occurrence of LHON and a multiple sclerosis-like disease (LHON-MS) is suggested to be 50 times more frequent than expected by chance [2], and has been considered a distinct entity named Harding’s disease [3]. With the exception of more frequent and severe involvement of the optic nerves LHON-MS seems to resemble relapsing remitting (RR) MS both clinically and radiologically [4], and most patients also have intrathecal synthesis of immunoglobulin G [2]. Immunomodulatory treatment such as natalizumab can therefore be considered. In MS, it is well established that disease activity returns within 4–7 months after discontinuation of natalizumab [5–7]. Some studies also reports possible rebound activity with disease activity exceeding pre-treatment levels or with particularly severe MS relapses [8–10]. Very little is known about the possible effects of starting and discontinuing potent immunomodulatory treatment in LHON-MS.

## Case presentation

The patient is born in 1965. Her first neurological symptom was a subacute right sided hemiparesis with partial recovery in 1989. In 1991 she experienced subacute and severe loss of vision on her left eye, without pain and with slow and incomplete recovery. The cerebrospinal fluid contained oligoclonal immunoglobulin G without counterpart in serum, and MS was diagnosed. In April 1992 she had subacute and pronounced loss of vision on her right eye, and 1 week later on the left eye (finger counting 0.5 m on both eyes). There has later been some fluctuation but no substantial improvement of her central visual acuity, whereas her peripheral vision has remained more intact. After some years her previously healthy son presented at the age of 16 years with bilateral painless sequential visual loss. Both pupils reacted to light. He had large absolute centrocecal scotomata. Funduscopy was unremarkable. No other neurological abnormalities were noted. He was treated with intravenous steroids followed by oral steroids without any benefit. LHON was suspected and mutation of G11778a in the ND4-gene was found. This finding resulted in further examination of our patient, and revealed that she is heteroplasmic (92 % mutant) for the same mutation.

Our patient reported transient ataxia with dysphagia in 1994, and ataxia with increased fatigue in 1999. Neurological examination in 2000 revealed spastic paraparesis and reduced vision (finger counting on both eyes), and interferon beta 1a (Rebif©) was initiated. She was seen yearly by an experienced MS neurologist who found that she was clinically stable the following years, with Expanded Disability Severity Scale (EDSS) score at 4.0. In June 2008 the patient cancelled the planned consultation because she felt she was clinically stable. She also had regular contact with an MS nurse, and in June 2009 she called the MS nurse and reported that she had suffered a possible mild attack with pain and transiently reduced strength in two fingers in her right hand, and that she wanted to discuss the possibility of switching the treatment to natalizumab (Tysabri©). In August the same year she again called the MS nurse and reported paresthesia in her left lower arm. These symptoms subsided after 4 weeks, again without subjective sequela. She was seen by her neurologist in October 2009, who found unchanged spastic paraparesis and reduced vision without evidence of new neurological deficits compared to previous examinations. She was able to walk 500 m without aid and EDSS was 4.0. MRI of the brain in November 2009 showed approximately 20 supra tentorial T2 lesions, the largest measuring 6 × 4 mm. There were no contrast enhancing lesions, and no lesions in the spinal cord. No previous MRI scans were available for comparison.

The neurologist concluded that she had likely suffered mild attacks without sequela in June 2008 and October 2009. For this reason, and also because the patient found the interferon injections increasingly intolerable, natalizumab 300 mg monthly was started in November 2009. The following years she reported no attacks, and yearly MRI was stable with numerous supratentorial lesions. In 2012 she tested weakly positive (JCV index 0.5) for John Cunningham virus antibodies (JCV Stratify©). The JCV index is a titer equivalent that predicts the risk of progressive multifocal leukoencephalopathy (PML) during natalizumab treatment. Weakly positive JCV indices (below 0.7) are estimated to be associated with a low PML risk (possibly up to 0.3 ‰) after 49–72 months treatment, compared to 17.0‰ in those with indices above 1.5 [11]. Neurological examination in both July 2014 and January 2015 revealed slightly increasing ataxia in the left arm and in both legs, although not to an extent that further affected gait distance (500 m) or EDSS (4.0). Because neurological disability had increased slightly in the absences of relapses since 2009, and because MRI scans in March 2012, April 2013 and October 2014 had not shown any new or gadolinium enhancing lesions, secondary progression was suspected. For this reason and because JCV index remained weakly positive (0.47 in January 2015), the last dose of natalizumab was given in January 2015. She could then walk 500 m without aid. MRI in April 2015 did not show new or gadolinium enhancing lesions. In the absence of clinical relapses or MRI activity, and because the progression was regarded as slow, no other disease modifying treatment was started.

**Fig. 1:**
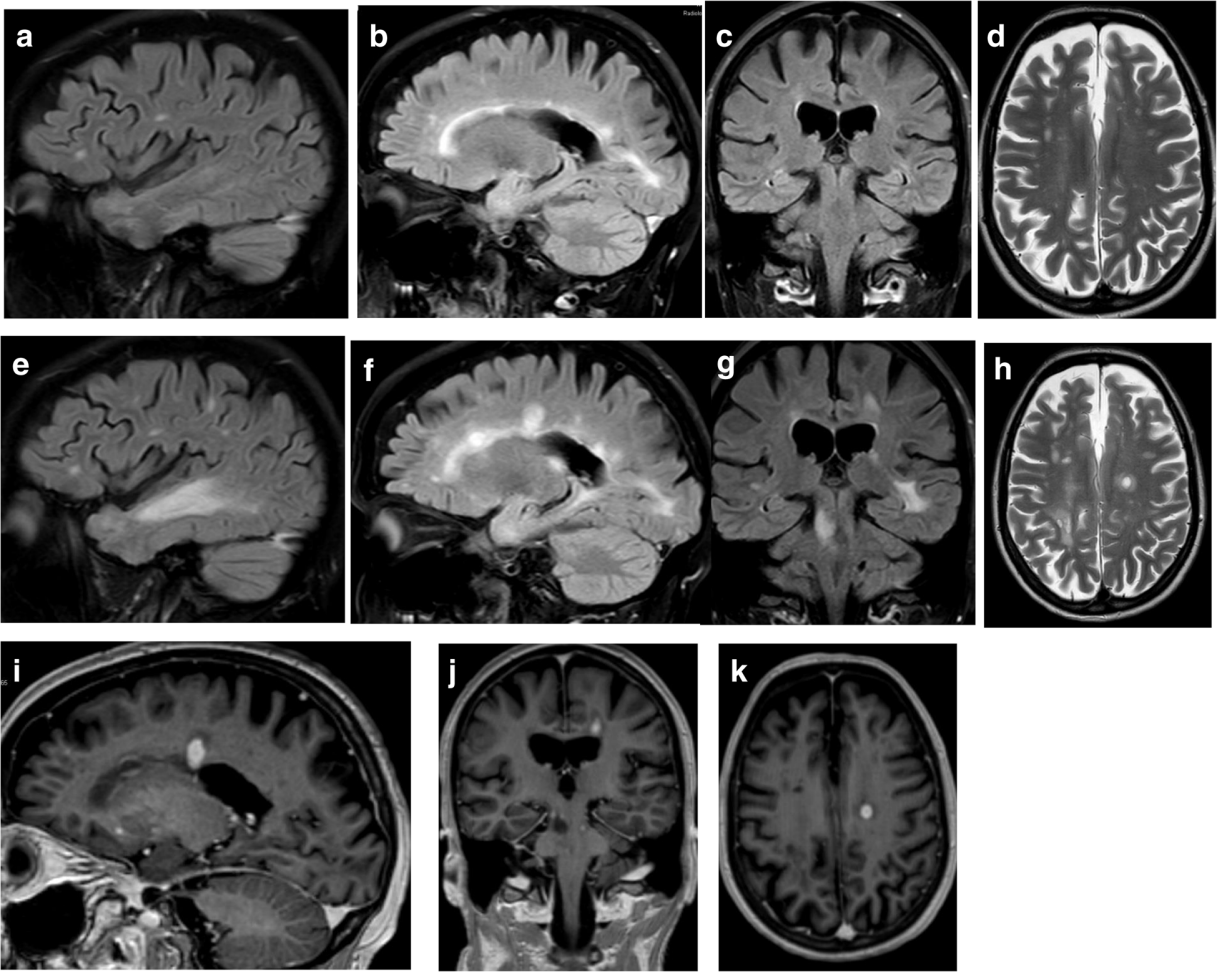
MRI during the early phase and at the peak of post-natalizumab exacerbation. The upper panel shows Fluid inversion recovery (FLAIR; **a**, **b** and **c**) and T2 (**d**) from June 2015. The middle panel shows examples of new and enlarging lesions on corresponding FLAIR (**e**, **f** and **g**) and T2 (**h**) sections at March 2016. The lower panel (**i**, **j** and **k**) shows examples of gadolinium enhancement on T1 images at March 2016

In June 2015 she was hospitalized with escalating gait difficulties and could only walk a few meters even with aid, corresponding to an estimated EDSS at 7.5. MRI of the brain and cervical and upper thoracic spinal cord did not show new or active lesions. She recovered somewhat (EDSS 6.0; corresponding to requirement of aid to walk 100 m), and no treatment was given. In December 2015 she reported subacute deterioration of vision at the left eye, with loss of finger counting. In March 2016 she was hospitalized with increasing left-sided hemiparesis and reduced sensation, urinary retention and dysphagia. Movement was restricted to wheelchair, and she could hardly raise her left arm against gravity. Compared to the last previous MRI from June 2015, MRI showed at least 12 new lesions in the cerebral hemispheres, two in pons, one in the left thalamus and several in the medulla (Fig. 1). Numerous lesions were active with gadolinium enhancement, including in the spinal cord from C6 to Th1. Some large and confluent lesions were susceptive of progressive multifocal leukoencaphalopathy. Her JCV index had increased to 1.25. A slight elevation of albumin index and pleocytosis (8 ﻿white blood cells/μl) was recorded in the first cerebrospinal fluid sample, but not after 14 days. PCR for JC virus in the cerebrospinal fluid was negative at both occasions. As PML was considered highly unlikely, and because the pronounced contrast enhancement both in the brain and in the medulla rather suggested severe and IRIS-like exacerbation of MS she was treated with high dose methylprednisolone for 5 days, followed by some clinical improvement of the hemiparesis, dysphagia and urinary retention. MRI 4 weeks after admission revealed marked regression of both confluent lesions and gadolinium enhancement in cerebrum and medulla. In agreement with the patient who has remained cognitively intact, treatment with alemtuzumab was started in April 2016. Her EDSS score peaked at 8.0 and was 6.5 in June and August 2016.

## Conclusions

This case story shows that severe disease activity resembling IRIS can occur after 27 years disease duration in a patient with LHON-MS, and raises the question whether this unusual exacerbation was triggered by the withdrawal of natalizumab 14 month previously.

The concept of rebound disease activity upon discontinuation of natalizumab in RRMS is not entirely clear. A systematic study of relapses in RRMS patients during the suspension of natalizumab in 2005 showed that disease activity returned to pre-natalizumab levels and peaked between 4 and 7 months, without evidence of rebound [6]. On the other hand, others have reported increased disease activity compared to pre-treatment levels in a substantial proportion of patients, including IRIS-like and lethal exacerbations [5]. In most cases the patients with possible rebound had aggressive RRMS prior to treatment with natalizumab. High risk of significant worsening after natalizumab withdrawal has, however, been reported also in patients with possible secondary progressive MS with EDSS exceeding four [10], which was one of the reasons for withdrawal in our patient.

Our patient had no MRI activity when the first clinical signs of disease activity appeared 6 months after discontinuation of natalizumab, and the full-blown IRIS-like exacerbation first appeared after 14 months. This is longer than reported in MS [5, 9, 12]. Saturation of alpha4 integrin is permissive for the immunological effect of natalizuamb. Desaturation to 50 % is likely to occur when serum levels of natalizumab fall below 1 μg/ml [13], which has been predicted to take almost 100 days in 50 % of MS patients on long-term treatment. Natalizumab can however be measured in the serum up to 260 days after the last infusion, with pronounced variation between individuals [14]. Interestingly, it has been demonstrated that natalizumab can exchange Fab-arms with wild-type IgG4 in natalizumab-treated patients, possibly contributing to observed variation in the elimination time [15]. It is thus possible that the time needed to desaturation of α4-integrin varies considerably between individuals, and that an observation period of up to 1 year after natalizumab withdrawal, as reported in several studies [6, 8, 10], is too short to capture all cases of possible rebound.

It must, however, also be considered whether the IRIS-like exacerbation in our patient was not causally related to the withdrawal of natalizumab. Because of the rarity, there are no systematic studies of the natural history of LHON-MS. With the exception of early and severe bilateral optic neuropathy, it does however not seem to differ very much from other patients with MS [2, 16]. Interestingly, the IRIS-like MRI findings in our patient share some features with another LHON-MS patient previously reported [17], who had not been treated with natalizumab or other immunomudulatory drugs, and who died after 19 years disease duration [18]. There are however differences, as the most prominent feature of the previously published patient was a cystic and space-occupying lesion in the frontal lobe and contrast enhancement seemed to be less prominent than in our patient.

It is not known if or how Leber mutations contribute to the MS-like pathology in LHON-MS, or how the condition should be treated. In contrast to previous reports [2], a recent study did not find more Leber mutations among MS patients than expected by chance [16], thus challenging that LHON-MS is a distinct entity. On the other side, mitochondrial dysfunction is indeed part of the pathogenesis in MS also in patients without LHON [19], and mitochondrial manganese superoxide has been reported to be upregulated in lesions outside the optic nerves in autopsy material from a patient with LHON-MS [18].

Given the radiological, immunological and clinical resemblance with MS, we find it reasonable to treat patients with LHON-MS with immunomodulatory drugs as other patients with MS. The current case report underscores the need to be cautious when considering withdrawal of natalizumab also in these patients, and suggests that severe rebound activity can occur more than a year after natalizumab withdrawal.

## Abbreviations

EDSS: Expanded Disability Status Scale
IRIS: Immunological reconstitution syndrome
LHON-MS: Leber’s hereditary optic neuropathy with multiple sclerosis

## References

[1] Rasool N, Lessell S, Cestari DM (2016). Leber hereditary optic neuropathy: bringing the lab to the clinic. Semin Ophthalmol.

[2] Palace J (2009). Multiple sclerosis associated with Leber's hereditary optic neuropathy. J Neurol Sci.

[3] Harding AE, Sweeney MG, Miller DH (1992). Occurrence of a multiple sclerosis-like illness in women who have a Leber's hereditary optic neuropathy mitochondrial DNA mutation. Brain.

[4] Matthews L, Enzinger C, Fazekas F (2015). MRI in Leber's hereditary optic neuropathy: the relationship to multiple sclerosis. J Neurol Neurosurg Psychiatry.

[5] Rasenack M, Derfuss T (2016). Disease activity return after natalizumab cessation in multiple sclerosis. Expert Rev Neurother.

[6] O'Connor PW, Goodman A, Kappos L (2011). Disease activity return during natalizumab treatment interruption in patients with multiple sclerosis. Neurology.

[7] Clerico M, Schiavetti I, De Mercanti SF (2014). Treatment of relapsing-remitting multiple sclerosis after 24 doses of natalizumab: evidence from an Italian spontaneous, prospective, and observational study (the TY-STOP Study). JAMA Neurol.

[8] Sorensen PS, Koch-Henriksen N, Petersen T, Ravnborg M, Oturai A, Sellebjerg F (2014). Recurrence or rebound of clinical relapses after discontinuation of natalizumab therapy in highly active MS patients. J Neurol.

[9] Larochelle C, Metz I, Lecuyer MA, et al. Immunological and pathological characterization of fatal rebound MS activity following natalizumab withdrawal. Mult Scler. 2016. doi:10.1177/1352458516641775. [Epub ahead of print]. 10.1177/1352458516641775 27037182

[10] Vidal-Jordana A, Tintore M, Tur C (2015). Significant clinical worsening after natalizumab withdrawal: Predictive factors. Mult Scler.

[11] Borchardt J, Berger JR (2016). Re-evaluating the incidence of natalizumab-associated progressive multifocal leukoencephalopathy. Mult Scler Relat Disord.

[12] Miravalle A, Jensen R, Kinkel RP (2011). Immune reconstitution inflammatory syndrome in patients with multiple sclerosis following cessation of natalizumab therapy. Arch Neurol.

[13] Khatri BO, Man S, Giovannoni G (2009). Effect of plasma exchange in accelerating natalizumab clearance and restoring leukocyte function. Neurology.

[14] Rispens T, Vennegoor A, Wolbink GJ, Polman CH, Killestein J (2012). Natalizumab remains detectable in patients with multiple sclerosis long after treatment is stopped. Mult Scler.

[15] Labrijn AF, Buijsse AO, van den Bremer ET (2009). Therapeutic IgG4 antibodies engage in Fab-arm exchange with endogenous human IgG4 in vivo. Nat Biotechnol.

[16] Pfeffer G, Burke A, Yu-Wai-Man P, Compston DA, Chinnery PF (2013). Clinical features of MS associated with Leber hereditary optic neuropathy mtDNA mutations. Neurology.

[17] Horvath R, Abicht A, Shoubridge EA (2000). Leber's hereditary optic neuropathy presenting as multiple sclerosis-like disease of the CNS. J Neurol.

[18] Kovacs GG, Hoftberger R, Majtenyi K (2005). Neuropathology of white matter disease in Leber's hereditary optic neuropathy. Brain.

[19] Lassmann H (2013). Pathology and disease mechanisms in different stages of multiple sclerosis. J Neurol Sci.

